# Case Report: A family of congenital cataract caused by a novel mutation in the CRYGC gene c.52G>A

**DOI:** 10.3389/fmed.2025.1624834

**Published:** 2025-07-24

**Authors:** Shuying Ma, Yao Lu, Xiao Shao, Qingyan Liu, Xin Yin, Min Xue

**Affiliations:** Graduate School, Bengbu Medical University; Department of Ophthalmology, Anhui No.2 Provincial People’s Hospital, Hefei, China

**Keywords:** congenital cataracts, CRYGC gene, whole exome sequencing, missense mutations, genetic disease

## Abstract

**Background:**

Congenital cataract refers to lens opacity present at birth or progressively developing in the neonatal period, caused by inherited genetic abnormalities or developmental disorders. The etiology of congenital cataract is multifactorial, and its exact pathogenesis remains incompletely understood. Generally, it can be broadly categorized into genetic factors and non-genetic factors (including environmental influences, intrauterine infections or complications during delivery). This case report presents a hereditary congenital cataract characterized by classical clinical manifestations, high penetrance, and a novel pathogenic gene mutation site that has not been previously documented in medical literature. We herein report this unique case to enhance our understanding of congenital cataract pathogenesis and expand the mutational spectrum associated with this ocular disorder.

**Case presentation:**

A 30-year-old female presented with blurred vision in her left eye since childhood. Slit-lamp microscopy revealed len opacity in the left eye. Based on the patient’s age of onset, ocular examination findings, and family history, the diagnosis of congenital cataract was established. Multiple family members had been previously diagnosed with “bilateral congenital cataracts.” Following cataract surgery, the patient’s visual acuity in the left eye improved to 20/40. To investigate the genetic etiology in this pedigree, whole-exome sequencing was performed on peripheral venous blood samples after obtaining informed consent from the patient and her family. Genetic testing identified a heterozygous missense mutation in exon 2 of the CRYGC gene (c.52G>A:p. Glu18Lys). This mutation results in the substitution of a highly conserved glutamic acid residue with lysine at position 18. Notably, this genetic variant was absent in unaffected family members.

**Conclusion:**

The patients in this pedigree exhibited an autosomal dominant inheritance pattern, with all affected individuals presenting bilateral lens opacities unaccompanied by systemic abnormalities, confirming a definitive diagnosis of congenital cataracts. Genetic screening identified a novel CRYGC gene mutation (c.52G>A:p. Glu18Lys) as the pathogenic cause of congenital cataracts in this family. Our findings expand the mutational spectrum of the CRYGC gene associated with congenital cataracts and provide enhanced insights into the molecular basis of this condition.

## Introduction

Congenital cataract is a blinding ocular disorder caused by abnormal lens metabolism leading to decreased transparency. Typical manifestations include visual acuity reduction, glare, and altered color perception. The lens opacities in congenital cataracts demonstrate diverse morphological patterns, including nuclear, anterior polar, posterior polar, punctate, disc-shaped, coraliform, coronary, and total cataract. With high incidence in neonates and considering the neonatal period as a critical window for visual development, early detection followed by successful cataract surgery remains the primary therapeutic approach to achieve optimal visual outcomes (1). Literature reports indicate that 30–50% of congenital cataracts are hereditary (2, 3), with inheritance patterns including autosomal dominant (AD), autosomal recessive (AR), and X-linked (XR) transmission. The pedigree we investigated conforms to an autosomal dominant inheritance pattern. This case report presents a novel mutation site in the pathogenic gene associated with autosomal dominant congenital cataract, highlighting the extensive heterogeneity and complexity of mutation loci in this condition. Our findings expand the mutational spectrum of genes implicated in congenital cataract pathogenesis, contributing to enhanced understanding of its genetic architecture.

## Case report

**Figure 1:**
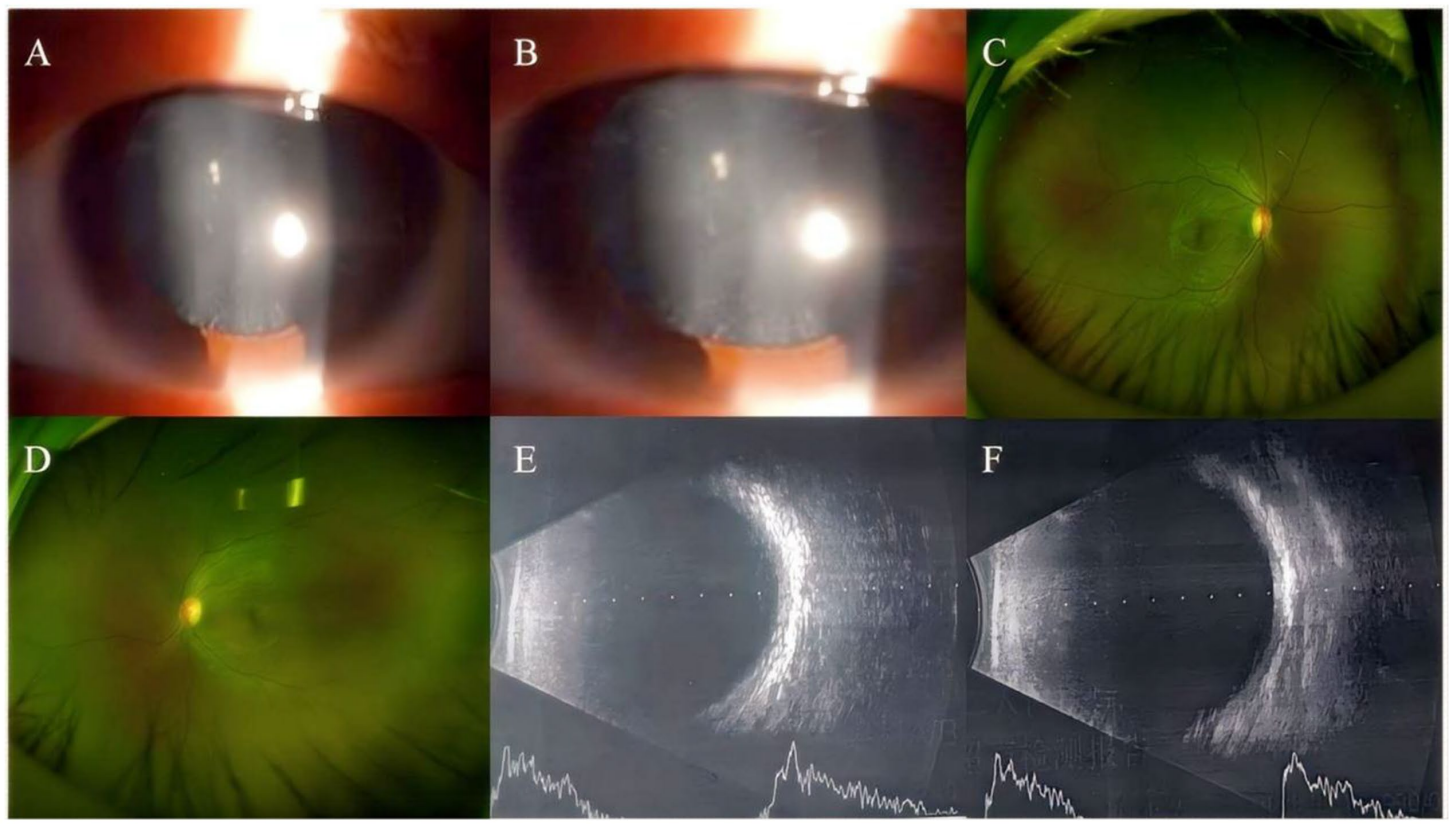
Anterior segment imaging. Anterior segment examination in the left eye of the proband reveals central cortical punctate opacities in the lens **(A,B)**. Ultra-widefield fundus imaging. Bilateral ultra-widefield fundus images of the proband demonstrate sharp margined optic disc with normal coloration, visible macular reflex, and attached retina (**C**: right eye, **D**: left eye). B-scan ultrasonography. Bilateral B-scan ultrasonography shows clear vitreous body and attached retina in both eyes (**E**: right eye, **F**: left eye).

**Figure 2:**
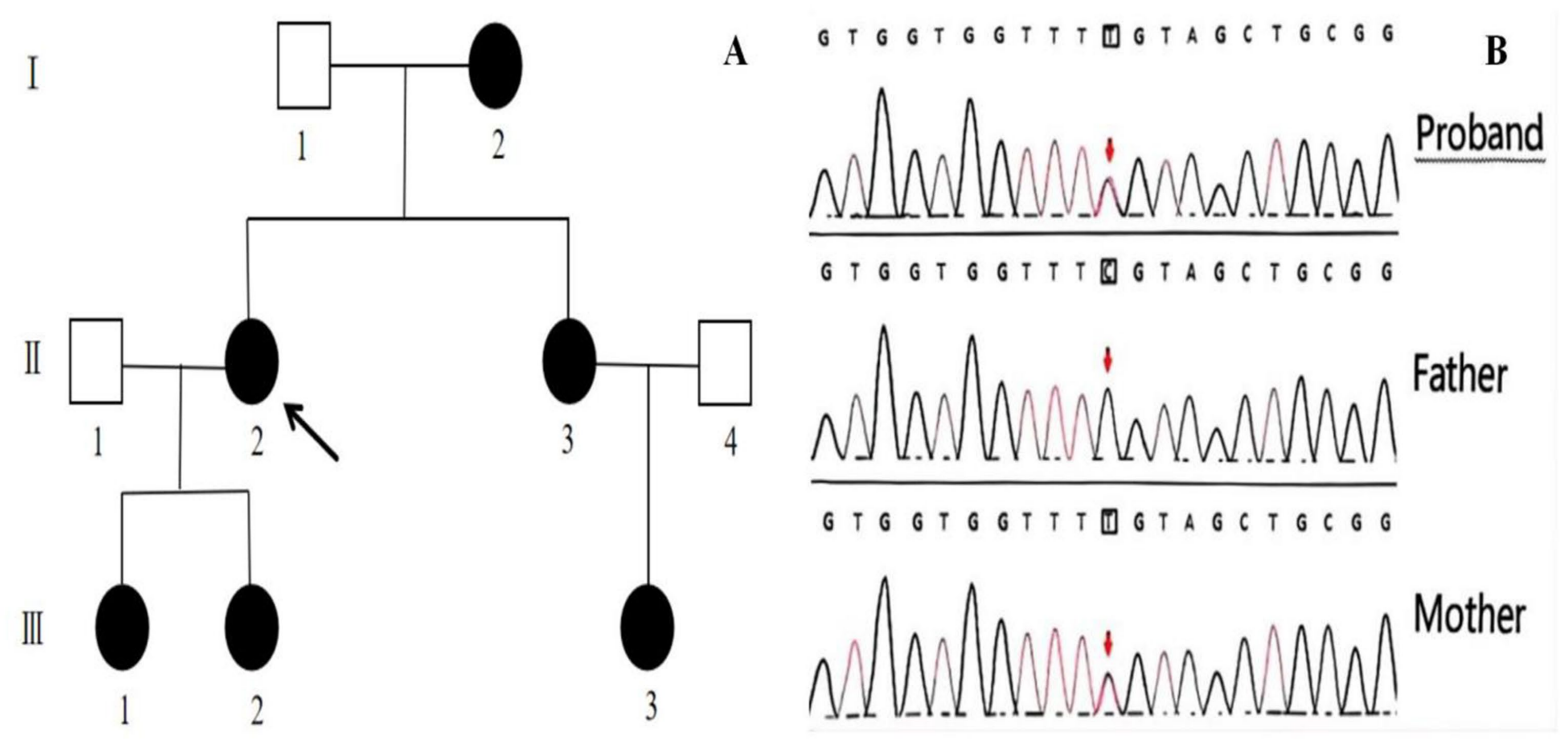
Genetic Pedigree of the family with congenital cataract **(A)**. The proband is indicated with an arrow. Gene sequencing results of the proband and her parents **(B)**. The sequencing results for the proband, her father, and her mother are displayed. Mutation site indicated by arrows. A heterozygous c.52G>A:p. Glu18Lys mutation in the CRYGC gene was identified in both the patient and the mother, while the father did not harbor this mutation.

**Figure 3:**
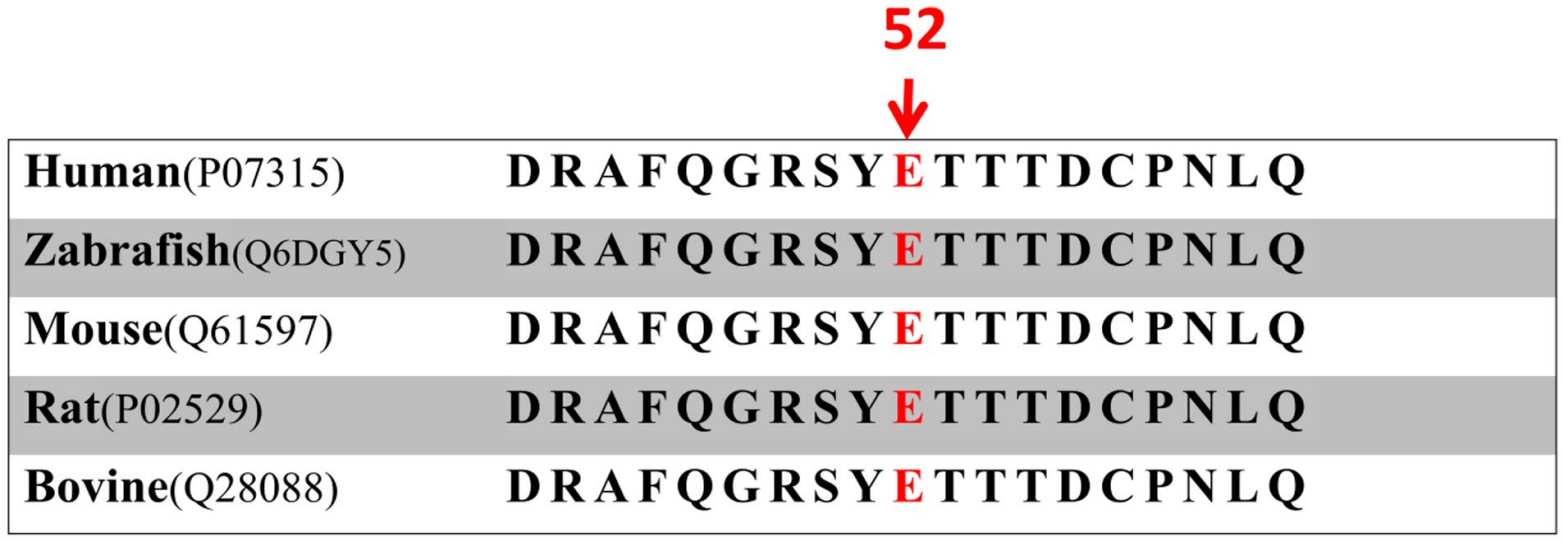
Comparative analysis of CRYGC gene conservation in humans and four mammalian species.

A 30-year-old Han Chinese female patient presented to the Anhui No.2 Provincial People’s Hospital in January 2024 with a poor vision in her left eye since childhood. The patient has undergone intraocular lens (IOL) implantation in the right eye. Ophthalmic examination: Best-corrected visual acuity (BCVA) was 20/40 in the right eye and 20/200 in the left eye. Non-contact tonometry (NCT) recorded intraocular pressures of 15 mmHg OD and 14 mmHg OS. Both eyes exhibited orthophoria with normal eyelid appearance. Corneas were clear bilaterally with anterior chambers of normal depth. Pupils were round, reactive to light (3 mm diameter). The right eye demonstrated a well-positioned intraocular lens (IOL), while the left eye revealed central cortical lens opacity. Funduscopic examination showed no significant abnormalities in either eye (Figure 1). Upon detailed inquiry into the family history, her mother had undergone bilateral cataract surgery due to bilateral congenital cataracts diagnosed during childhood. The patient’s father had an unremarkable physical examination. There was no history of prenatal drug exposure or radiation contact during pregnancy. Multiple family members across generations had been diagnosed with “bilateral congenital cataracts,” all presenting with poor binocular vision since childhood. Clinical manifestations included punctate cortical opacities in the central lens with normal fundus, without associated nystagmus or strabismus (Figure 2A; Table 1). Based on these findings, this female patient was clinically diagnosed as “congenital cataract in the left eye and intraocular lens implantation in the right eye.” To further investigate the genetic etiology, whole exome sequencing was performed using peripheral venous blood samples with informed consent from the patient and the family. The results revealed a heterozygous missense mutation in exon 2 of the CRYGC gene: c.52G>A:p. Glu18Lys. This mutation leads to the substitution of a highly conserved glutamic acid residue with lysine at position 18 of the CRYGC protein. Although genetic testing was not performed on all affected family members, their clinical history, symptoms, and signs definitively support a diagnosis of bilateral congenital cataracts. Furthermore, the characteristics of their lens opacities were identical to those of the proband, presenting as central cortical punctate opacities (Figure 2B). These findings confirm that the CRYGC c.52G>A:p. Glu18Lys mutation constitutes the genetic pathogenesis underlying congenital cataracts in this family. To evaluate evolutionary constraints, we performed a cross-species conservation analysis of CRYGC between humans and four Mammalian animal species: Human, Zabrafish, Mouse, Rat and Bovine, confirms high functional preservation of CRYGC gene (Figure 3). We further evaluated the pathogenicity of the CRYGC mutation c.52G>A:p. Glu18Lys using the following established web-based prediction tools: PROVEAN, SIFT, MutationTaster and PolyPhen-2. This change was assessed to be deleterious (PROVEAN score = −3.505) by analysis with the PROVEAN online tool,^1^ to be not tolerated by SIFT,^2^ to be probably damaging by PolyPhen-2 with a score of 0.998,^3^ and to be disease causing by mutation taster^4^ (Table 2). Following cataract surgery, the patient’s visual acuity in the affected eye recovered to 20/40 postoperatively.

**Table 1:** Clinical manifestations of patients in this congenital cataract pedigree.

Patients	Sex	Age	Sight (Right/Left)	Lens	Nystagmus	Fundus
I2	Female	53	20/100;20/100	IOLs *in situ*; Bilateral mild posterior capsular opacification	No	Normal
II2	Female	30	20/40;20/200	Right eye: IOL in situ. Left eye: punctate opacity in the central cortex of the lens.	No	Normal
II3	Female	28	20/100;20/40	Bilateral punctate opacities in the central cortex of the lenses	No	Normal
III1	Female	10	20/25;20/40	Bilateral punctate opacities in the central cortex of the lenses	No	Normal
III2	Female	2	Unknow	Bilateral punctate opacities in the central cortex of the lenses	No	Normal
III3	Female	6	20/40;20/40	Bilateral punctate opacities in the central cortex of the lenses	No	Normal

**Table 2:** CRYGC mutation (c.52G>A) results from pathogenicity prediction software.

Results	PROVEAN	SIFT	Polyphen-2	Mutation taster
Score	−3.505	0.000	0.998	0.999
Prediction result	Deleterious	Affect protein function	Probably damaging	Disease causing

## Discussion

Congenital cataract stands as one of the most prevalent causes of visual impairment and blindness in children worldwide. According to a systematic review, the global incidence ranges from approximately 1.91 to 4.24 per 10,000 live births, with developing countries showing a significantly higher rate of up to 7.43 per 10,000 (4). Although being a treatable cause of blindness, congenital cataract frequently occurs during infancy and within the first postnatal year, often goes undetected leading to missed diagnoses, and consequently results in irreversible visual damage. Blurred vision since early childhood typically serves as the chief complaint at initial presentation. However, with disease progression, complications may emerge including leukocoria, nystagmus, amblyopia, strabismus, diplopia, myopia, and altered color perception. The pathogenesis of congenital cataract is complex, with genetic factors accounting for approximately one-quarter to one-half of cases (5, 15). Most hereditary forms follow an autosomal dominant inheritance pattern, though autosomal recessive and X-linked inheritance have also been documented (6). Advances in molecular genetics have identified over 50 genes and loci associated with congenital cataract, including crystallin genes (CRYAA, CRYAB, CRYBA1/A3, CRYBB1, CRYBB2), membrane transport protein genes (GJA3, GJA8, MIP), transcription regulator genes (HSF4, PITX3, MAF), and cytoskeletal protein genes (BFSP2), among others (7). Notably, crystallin gene mutations account for approximately 50% of reported familial cases of hereditary congenital cataracts. To date, more than 300 distinct mutation sites in crystallin genes have been identified as causative factors for congenital cataracts (8).

The lens contains crystallins, structural proteins characterized by high solubility and stability, which are essential for maintaining lens transparency and high refractive index (9). The crystallin family comprises α-, β-, and γ-crystallins, collectively accounting for 90% of lens proteins. Among these, γ-crystallins play a pivotal role in preserving crystalline transparency (10, 11). The γ-crystallin family consists of eight subtypes encoded by CRYGA, CRYGB, CRYGC, CRYGD, CRYGE, CRYGF, CRYGS, and CRYGN genes. The CRYGC gene and its protein product are closely associated with various ocular pathologies, particularly cataractogenesis. Located at chromosome 2q33.3, the CRYGC gene spans 1.9 kb with three exons, encoding a 173-amino acid protein with molecular weight of 21 kDa. Mutations in the CRYGC gene disrupt the structural stability of γ-crystallin, inducing protein misfolding, compromising its solubility, and triggering abnormal aggregation. These aggregates ultimately act as light-scattering centers, culminating in the development of congenital cataracts. The score pathogenic process is classified as a protein conformational disorder. Current research priorities include: (1) Elucidating the intricate mechanisms underlying protein aggregation (e.g., chaperone interactions, liquid–liquid phase separation - LLPS); (2) Exploring novel therapeutic avenues, including gene therapy and small-molecule drug interventions; (3) Refining genotype–phenotype correlations; (4) Leveraging cutting-edge technologies (e.g., gene-editing tools like CRISPR/Cas9, advanced imaging modalities) to establish more physiologically relevant and sophisticated models for mechanistic studies. Zhong et al. (12) identified 17 CRYGC mutations in Chinese pedigrees, including six missense mutations (p. Thr5Pro, p. Arg48His, p. Gly129Cys, p. Ser166Phe, p. Arg168Trp, and p. Tyr46Asp), five nonsense mutations (p. Cys109X, p. Tyr139X, p. Trp157X, p. Tyr144X, and p. Arg169X), and six frameshift mutations (p. Gly41insfsX62, p. Cys42AlafsX60, p. Gln55ValfsX50, p. Asp65ThrfsX38, p. Arg142GlyfsX5, and p. Arg142AlafsX22). Additional mutations (p. Gly129Cys and p. Trp131Arg) have been reported by Zhou et al. (13) and Delas et al. (14). Notably, the c.52G>A:p. Glu18Lys heterozygous missense mutation identified in our case represents a novel variant not previously documented in the ClinVar database.

## Conclusion

We report a familial case of congenital cataract and performed comprehensive genetic analysis using whole exome sequencing. The pedigree exhibited characteristics consistent with autosomal dominant inheritance, demonstrating distinctive punctate opacities in the central lens cortex in affected family members. Genetic investigation revealed a novel pathogenic mutation in the CRYGC gene (c.52G>A:p. Glu18Lys). Our findings expand the mutational spectrum of congenital cataracts and provide new insights into its etiology and prenatal diagnosis. However, the pathogenic mechanisms underlying the CRYGC c.52G>A:p. Glu18Lys mutation in congenital cataract development require further investigation through functional studies.

## Data Availability

The original contributions presented in the study are included in the article, further inquiries can be directed to the corresponding author.

## Footnotes

^1^ http://provean.jcvi.org/index.php

^2^ http://sift.bii.a-star.edu.sg/index.html

^3^ http://genetics.bwh.harvard.edu/pph2/index.shtml

^4^ http://www.mutationtaster.org/
